# Variations of Epstein-Barr Virus Nuclear Antigen 1 in Epstein-Barr Virus-Associated Gastric Carcinomas from Guangzhou, Southern China

**DOI:** 10.1371/journal.pone.0050084

**Published:** 2012-11-26

**Authors:** Jian-ning Chen, Na-na Zhang, Ye Jiang, Da-yang Hui, Zi-jin Wen, Hai-gang Li, Yun-gang Ding, Hong Du, Chun-kui Shao

**Affiliations:** 1 Department of Pathology, The Third Affiliated Hospital, Sun Yat-sen University, Guangzhou, People’s Republic of China; 2 Department of Pathology, The Second Affiliated Hospital, Sun Yat-sen University, Guangzhou, People’s Republic of China; 3 State Key Laboratory of Ophthalmology, Zhongshan Ophthalmic Center, Sun Yat-Sen University, Guangzhou, People’s Republic of China; 4 Department of Pathology, Guangzhou First Municipal People’s Hospital, Guangzhou, People’s Republic of China; Kagoshima University Graduate School of Medical and Dental Sciences, Japan

## Abstract

Epstein-Barr virus (EBV) nuclear antigen 1 (EBNA1) is the only viral protein consistently expressed in all EBV-associated malignancies, and play a critical role in the onset, progression, and/or maintenance of these tumors. Based on the signature changes at amino acid residue 487, EBNA1 is classified into five distinct subtypes: P-ala, P-thr, V-leu, V-val and V-pro. In the present study, the sequence variations of EBNA1 in EBV-associated gastric carcinoma (EBVaGC) and throat washing (TW) samples of healthy EBV carriers in Guangzhou, southern China, where nasopharyngeal carcinoma (NPC) is endemic, were analyzed by PCR and DNA sequencing. V-val subtype was the most predominant (53.6%, 15/28) in EBVaGC, followed by P-ala (42.9%, 12/28) and V-leu (32.1%, 9/28) subtypes. In TWs of healthy EBV carriers, V-val subtype was also predominant (85.7%, 18/21). The sequence variations of EBNA1 in EBVaGC were similar to those in TW of healthy EBV carriers (*p*>0.05), suggesting that the EBV strains in EBVaGC might originate from the viral strains prevalent within the background population. The predominance of V-val subtype in EBVaGC in Guangzhou was similar to that in EBVaGC in northern China and Japan, but was different from that in EBVaGC in America, suggesting that the variations of EBNA1 in EBVaGC represent geographic-associated polymorphisms rather than tumor-specific mutations. In addition, the EBNA1 variations in EBVaGC in gastric remnant carcinoma were also determined. V-leu subtype was detected in all 4 (100%) cases, although 2 cases occurred as mixed infection with P-ala subtype. This is different from the predominant V-val subtype in EBVaGC in conventional gastric carcinoma, suggesting that V-leu might be a subtype that adapts particularly well to the microenvironment within the gastric stump and enters the remnant gastric mucosa epithelia easily. This, to our best knowledge, is the first investigation of EBNA1 polymorphisms in EBVaGC from endemic area of NPC.

## Introduction

Epstein-Barr virus (EBV) is a lymphotrophic virus that infects over 90% of the adults worldwide and is closely associated with lymphoid neoplasms, such as Burkitt’s lymphoma (BL), Hodgkin lymphoma (HL) and B-cell lymphomas among immunosuppressed patients [1], [2]. EBV has also been suspected to cause epithelial malignancies such as nasopharyngeal carcinoma (NPC) and a subset of gastric carcinoma defined as EBV-associated gastric carcinoma (EBVaGC) [3]. EBVaGC represents about 10% of gastric carcinoma worldwide; however, the proportion varies from country to country and ranges from 1.3% to 20.1% [4], [5], [6]. Our previous study demonstrated that the proportion of EBVaGC in gastric carcinoma in Guangzhou, southern China was 6.7% (45/676) [7].

After infection, EBV persists in host in latency cycle and constitutively expresses a limited set of viral gene products, the so-called latent products, which comprise six EBV nuclear antigens (EBNAs 1, 2, 3A, 3B, 3C and -LP), three latent membrane proteins (LMPs 1, 2A and 2B) and two EBV-encoded small non-coding RNAs (EBERs 1 and 2) [1]. Three latency types have been described depending on which of these latent products are expressed. Latency I is limited to only EBERs and EBNA1 expression; latency II includes LMP1 and 2 in addition; and latency III is defined by expression of EBERs, all six EBNA proteins and two LMP proteins [8]. Among them, EBNA1 is the only viral protein consistently expressed in all EBV-associated malignant tissues [9]. EBNA1 is essential for the persistence and replication of the EBV genome in latently infected cells [10], [11], [12]. Recently, it is shown that EBNA1 induces genomic instability and reactive oxygen species (ROS)-mediated DNA damage response [13], [14]. These findings suggest that EBNA1 may play a critical role in the onset, progression, or maintenance of these tumors [15], and may act as a potential oncogene [16].

The EBNA1 protein is composed of unique amino-terminal (residues 1∼89) and carboxyl-terminal (residues 327∼641) domains flanking a large Gly-Ala repeat (residues 90∼326) [17]. The Gly-Ala repeat domain of EBNA1 prevents proteasome-dependent processing for presentation of EBNA1 on major histocompatibility complex (MHC) class I, and makes it elusive to CD8+ cytotoxic T lymphocytes (CTLs) [18], [19]. In the amino- and carboxyl-termini, several function domains have been identified, including chromosome binding domain (amino acids [aa] 33∼89), dimerization domain (aa 501∼532 and aa 554∼598), DNA binding domain (aa 459∼487), and transactivation domain (aa 450∼641) [20], [21], [22], [23].

As previously reported, based on the signature changes at aa residue 487 in the carboxyl-terminal of EBNA1, EBNA1 has been classified into five distinct subtypes, including prototype B95.8 strain sequence P-ala, a closely related subtype P-thr, and three more distant variants V-leu, V-val and V-pro [24], [25]. To date, there were only two papers concerning about the EBNA1 variations in EBVaGC. Chen *et al.* [26] examined the carboxyl-terminal sequence of EBNA1 in EBVaGC from two different ethnic populations. They found that all 25 (100%) cases of EBVaGC from Japan harbored the exclusive V-val subtype, while in 17 EBVaGC cases from America, P-thr subtype was predominant, accounting for 52.9% (9/17) of the cases. Only 1 (5.9%) case was of V-val subtype. Wang *et al.* [27] investigated the EBNA1 variations in 41 EBVaGCs from Northern China, and found that V-val subtype was prevalent in EBVaGCs, accounting for 78.1% (32/41) of the cases. Japan, America and northern China are non-endemic areas of NPC. The EBNA1 variations in EBVaGC in the endemic area of NPC have not been investigated yet.

Guangzhou, located in southern China, is well known as the high-incidence area of NPC in the world [3]. NPC is known as an EBV-associated epithelial malignancy. In addition, a special EBV variant, variant-type “f”, is predominant in NPC and is strongly associated with NPC in Guangzhou [28], [29]. In our previous study [7], we found that the predominant EBV variant in EBVaGC in Guangzhou was prototype F, which is different from that in NPC in this area. Moreover, a new identified variant, mut-W1/I1 variant, which shows a T to C mutation at position 148,972 (wild type EBV coordinates), was found in the majority of the EBVaGCs in Guangzhou. However, this mutation could not be found in the NPC-derived EBV stain GD1 [30]. These provide evidence that there may be a disease-related association between EBV variants, at least in EBVaGC versus NPC in patients drawn from the same population. Thus, the sequence variations of EBNA1 in EBVaGC in Guangzhou were of our interest. It may be of help in clarifying the pathogenic roles of EBV in epithelial malignancies to compare the EBNA1 variations in EBVaGC in Guangzhou with those in NPC and in healthy EBV carrier in the same area and those in EBVaGC in other areas.

Gastric remnant carcinoma (GRC) was originally defined as a carcinoma occurring in the gastric stump at least 5 years after a distal gastrectomy for benign diseases such as gastric ulcer and duodenal ulcer [31], [32]. Subsequently, it has been used to define all carcinomas arising from the remnant stomach after partial gastrectomy, regardless of the initial disease or operation, and includes local recurrence in the gastric stump after partial gastrectomy for gastric carcinoma [33], [34]. In our previous studies, we found that the proportion of EBVaGC in GRC was significantly higher than that in conventional gastric carcinoma (CGC) which occurs in the intact stomach in Guangzhou (30.8% vs. 6.7%) [7], [35]. Similar findings were also reported by other groups in Japan (27.1% vs. 6.4%) [36], Korea (29% vs. 6%) [37] and Netherlands (35% vs. 8%) [38]. Are the EBNA1 sequence variations in EBVaGC in GRC different from those in EBVaGC in CGC? To date, no study on polymorphisms of EBNA1 in EBVaGC in GRC is available.

Therefore, in the present study, the sequence variations of EBNA1 in EBVaGC in CGC, EBVaGC in GRC and throat washing (TW) samples of healthy EBV carriers in Guangzhou, sorthern China, where NPC is endemic, were investigated. We also compared the EBNA1 variations in EBVaGC with those in NPC and healthy donors in the same area and those in EBVaGC in other areas of the world, in order to explore the association between sequence variations of EBNA1 and EBVaGC, and to clarify the issue whether EBNA1 subtypes are disease associated or geographically distributed.

## Results

### Clinicopathologic Characteristics of EBVaGC in CGC and GRC

In the present study, 53 cases of EBVaGC, including 45 (84.9%) cases of CGC and 8 (15.1%) cases of GRC, were investigated. The clinicopathologic characteristics of EBVaGC in CGC and GRC are summarized in Table 1. The mean age was 51.5±13.9 years (range: 23–76 years) for the EBVaGC cases in CGC, and 67.6±7.4 years (range: 55–79 years) for the EBVaGC cases in GRC. All cases were advanced gastric carcinomas.

**Table 1:** Clinicopathologic characteristics of EBVaGC in CGC and GRC.

Variables	Total*	EBVaGC in CGC	EBVaGC in GRC	*p* †
Gender				0.333
Male	45	37	8	
Female	8	8	0	
Age (years)				0.002
≤40	11	11	0	
40∼60	25	24	1	
>60	17	10	7	
Mean ± SD‡		51.5±13.9	67.6±7.4	0.000
Macroscopic type§				0.581
1	8	7	1	
2	18	16	2	
3	21	18	3	
4	6	4	2	
Location§				
Cardia	15	15	/	
Body	14	14	/	
Antrum	14	14	/	
Whole||	2	2	/	
Stump	8	/	8	
Histology¶				0.333#
Intestinal	8	8	0	
pap	0	0	0	
tub1	0	0	0	
tub2	8	8	0	
Diffuse	45	37	8	
por1	12	12	0	
por2	33	25	8	
sig	0	0	0	
muc	0	0	0	
Invasion§				1.000
T1	0	0	0	
T2	4	4	0	
T3	42	35	7	
T4	7	6	1	
Stage (pTNM)§				0.424
1a	0	0	0	
1b	1	1	0	
2	11	8	3	
3a	19	15	4	
3b	8	8	0	
4	14	13	1	

* Total number of gastric carcinoma cases in each group.

† *p*-values were obtained from Fisher’s exact tests or Student’s *t* tests.

‡ SD: standard deviation.

§ Japanese classification.

|| Cases involved the whole stomach.

¶ Lauren classification and Japanese classification.

# Lauren classification.

### EBNA1 Expressed in 93.3% EBVaGCs in CGC and 87.5% EBVaGCs in GRC

**Figure 1:**
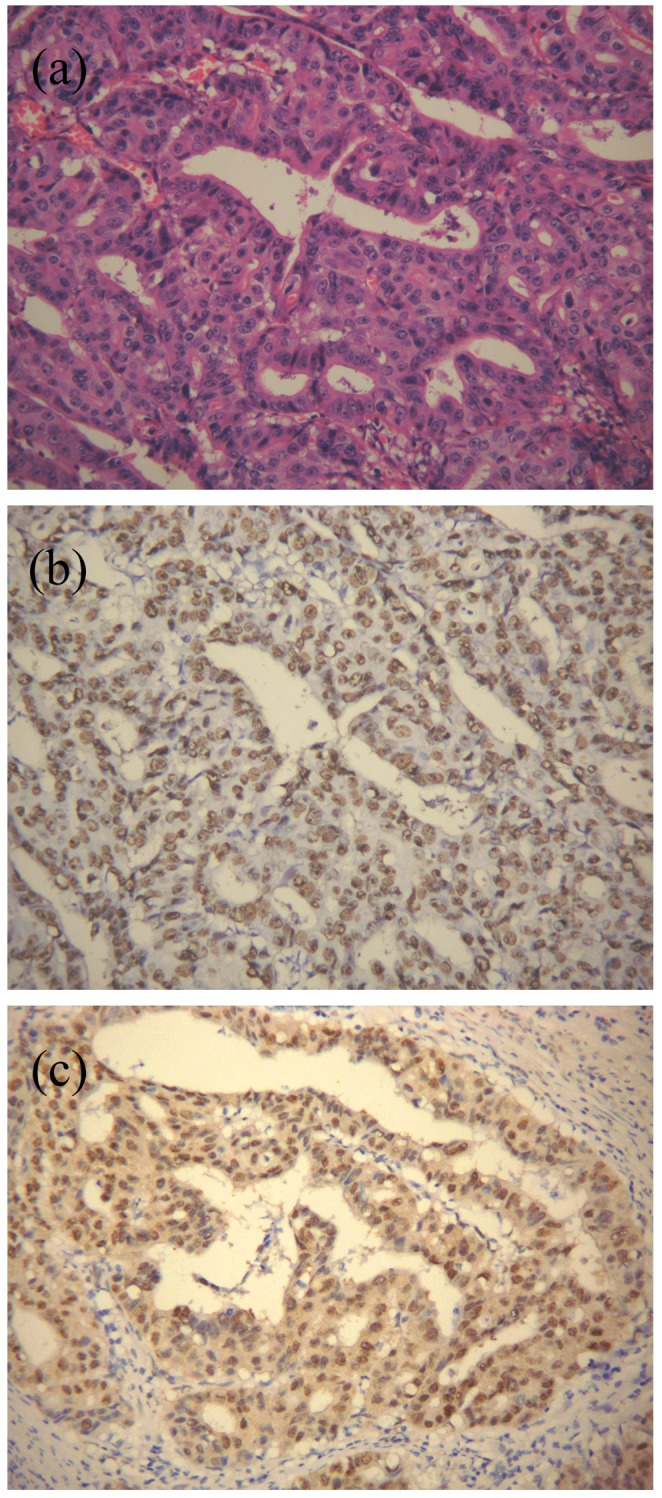
Expression of EBNA1 in EBVaGC. (a) *H&E* staining of a moderate differentiated tubular gastric adenocarcinoma. The histology of this case was intestinal-type carcinoma according to the Lauren classification. (b) EBER-1 *in situ* hybridization of the same case. The EBER-1-positive signals were restricted only to the tumor nuclei but not in surrounding non-tumor cells. (c) Immunohistochemistry staining for EBNA1 of the same case. Positive staining can be seen in almost all tumor nuclei. No positive staining was detected in infiltrating lymphocytes in and around the tumor nests. (Original magnification ×200).

The immunostaining for EBNA1 was successfully performed in all 53 EBVaGC cases. Forty-two (93.3%) of the 45 EBVaGC cases in CGC and 7 (87.5%) of 8 EBVaGC cases in GRC displayed diffuse positive signals in the nuclei of the tumor cells (Figure 1). There was no correlation between EBNA1 expression and the clinicopathologic parameters of EBVaGC, which include the age and sex of the patient, the location, histology and stage of the tumor (all *p*>0.05, data not shown).

### V-val Subtype was Predominant in EBVaGCs in CGC

**Figure 2:**
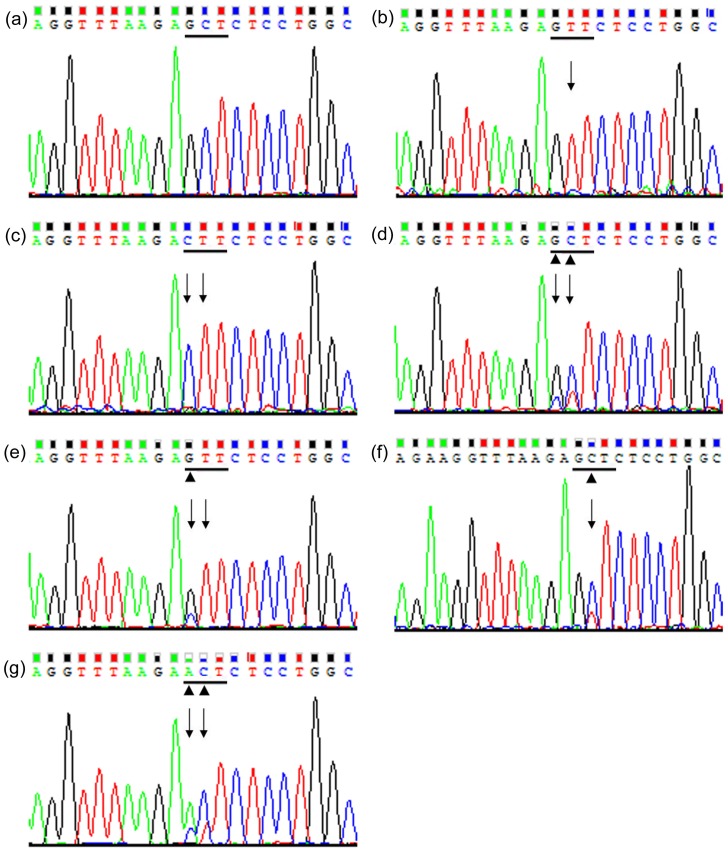
Sequence determination of EBNA1 subtypes. EBNA1 is classified into five subtypes, P-ala, P-thr, V-val, V-leu, and V-pro according to the polymorphism at the signature amino acid residue 487 (coded by nucleotides 109,408∼109,410). Partial sequences of the EBNA1 gene (nucleotides 109,398∼109,418) are shown and nucleotides 109,408∼109,410 are underlined. (a) P-ala subtype. P-ala was determined when the sequence was identical to B95.8 prototype. From nucleotides 109,408∼109,410 P-ala subtype showed GCT singals. (b) V-val subtype. V-val was confirmed when C signal changed to T at nucleotide 109,409. (c) V-leu subtype. V-leu was confirmed when GCT signals changed to CTT at nucleotides 109,408∼109,410. (d) Co-infection of P-ala and V-leu subtypes. G and C signals occurred simultaneously at nucleotide 109,408, and C and T signals occurred simultaneously at nucleotide 109,409. (e) Co-infection of V-val and V-leu subtypes. Co-infection of V-val and V-leu was defined when G and C signals occurred simultaneously at nucleotide 109,408, and TT signals at nucleotides 109,409∼109,410. (f) Co-infection of P-ala and V-val subtypes. C and T signals occurred simultaneously at nucleotide 109,409, and G and T signals at nucleotides 109,408 and 109,410, respectively. (g) Co-infection of V-leu and P-thr subtypes. A and C signals occurred simultaneously at nucleotide 109,408, and C and T signals occurred simultaneously at nucleotide 109,409. The arrows show mutated nucleotides. Dual signals occurred simultaneously at the nucleotides are indicated with solid triangles.

EBNA1 fragment was successfully amplified and sequenced in 28 (62.2%) EBVaGCs in CGC. All nucleotide variations were identified by comparing with the B95.8 prototype sequence. The sequencing results showed the presence of a single EBNA1 sequence in 19 (67.9%) of the 28 cases, whereas the remaining 9 (32.1%) samples displayed dual EBNA1 sequences. The distribution of EBNA1 subtypes in EBVaGC in CGC is illustrated in Figure 2 and Table 2. The V-val subtype was the most predominant (53.6%, 15/28) in EBVaGCs in CGC, followed by P-ala (42.9%, 12/28) and V-leu (32.1%, 9/28) subtypes. All the tumor samples which harbored the V-val subtype showed the same clustered point mutations. They included 8 nucleotide mutations, leading to 7 amino acid substitutions. The tumor samples harbored the V-leu subtype also showed the same clustered point mutations, which included 12 nucleotide mutations, leading to 10 amino acid substitutions. The nucleotide sequence variations as well as the amino acid changes of different EBNA1 subtypes were shown in Table 3.

**Table 2:** Distribution of EBNA1 subtypes in EBVaGC in CGC, EBVaGC in GRC and TWs of healthy EBV carriers from Guangzhou, southern China.

EBNA1 subtype(s)	EBVaGC in CGC No. (%)	EBVaGC in GRC No. (%)	TWs of healthy EBV carriers No. (%)
V-val	10 (35.7)	0 (0)	15 (71.4)
P-ala	7 (25)	0 (0)	1 (4.8)
V-leu	2 (7.1)	2 (50)	0 (0)
P-ala & V-leu	3 (10.7)	2 (50)	2 (9.5)
V-val & V-leu	3 (10.7)	0 (0)	1 (4.8)
P-ala & V-val	2 (7.1)	0 (0)	2 (9.5)
V-leu & P-thr	1 (3.6)	0 (0)	0 (0)
Total	28 (100)	4 (100)	21 (100)

**Table 3:** EBNA1 sequence variations of different EBNA1 subtypes in EBVaGC in CGC.

EBNA1 subtype	439	471	475	476	479	487	492	499	500	502	520	524	525	528	533
P-ala	GCA	CAA	AAC	CCG	GAG	GCT	AGT	GAC	GAA	ACT	CTA	ACT	GCC	ATT	CTT
	A	Q	N	P	E	A	S	D	E	T	L	T	A	I	L
V-val	ACA					GTT		GAG		AAT	CTC	ATT		GTT	ATT
	T					V		E		N	L	I		V	I
V-leu		GAA	AGC	CAG		CTT	TGT	GAG	GAT	AAT	CTC	ATT	GGC		
		E	S	Q		L	C	E	D	N	L	I	G		
P-thr				CAG	CAG	ACT	TGT	GAT			CTC	ATT			
				Q	Q	T	C	D			L	I			

The nucleotide sequence variations as well as the amino acid changes in the sequenced part of the carboxyl-terminus (codons 431–540) of the EBNA1 gene are shown. Numbers across the top correspond to the amino acid positions. For P-ala subtype, all coding triplets and amino acids are listed. For other subtypes, only coding triplets affected by single base mutations and corresponding amino acids are indicated. Mutated nucleotides are underlined. Amino acids are identified by one-letter codes.

### V-val Subtype was Associated with TNM Staging of EBVaGC in CGC

We separated the 28 EBVaGC cases as two groups. One is V-val group (15 cases), which harbored the V-val subtype, including infection with signal V-val subtype and co-infection with V-val subtype and other subtypes. The other is non-V-val group (13 cases), which harbored other subtype(s) rather than V-val subtype. In the V-val group, there were 5 cases in TNM stage I and II, and 10 cases in stage III and IV, respectively. In the non-V-val group, all the 13 cases were in TNM stage III and IV. There was a significant difference between V-val group and non-V-val group with regard to TNM staging (*p* = 0.044). The detailed clinicopathologic data and the EBNA1 subtype(s) of the 28 EBVaGC cases are showed in Table S1.

### V-val Subtype was Predominant in EBVaGCs without Prominent Lymphoid Stroma

We classified the 28 EBVaGC cases into two groups: EBVaGCs with prominent lymphoid stroma (10 cases) and EBVaGCs without prominent lymphoid stroma (18 cases). P-ala subtype was predominant (60%, 6/10) in EBVaGCs with prominent lymphoid stroma, whereas V-val subtype was prevalent (61.1%, 11/18) in EBVaGCs without prominent lymphoid stroma. The distribution of EBNA1 subtypes in EBVaGCs with and without prominent lymphoid stroma is showed in Table 4.

**Table 4:** Distribution of EBNA1 subtypes in EBVaGC with and without prominent lymphoid stroma.

EBNA1 subtype(s)	EBVaGC with prominent lymphoid stroma No. (%)	EBVaGC without prominent lymphoid stroma No. (%)
V-val	2 (20)	8 (44.4)
P-ala	4 (40)	3 (16.7)
V-leu	1 (10)	1 (5.6)
P-ala & V-leu	1 (10)	2 (11.1)
V-val & V-leu	1 (10)	2 (11.1)
P-ala & V-val	1 (10)	1 (5.6)
V-leu & P-thr	0 (0)	1 (5.6)
Total	10 (100)	18 (100)

### No V-val Subtype but Predominant V-leu Subtype was Detected in EBVaGCs in GRC

EBNA1 fragment was also successfully amplified and sequenced in 4 (50%) out of the 8 EBVaGCs in GRC. The EBNA1 sequences in two cases were V-leu subtype, while those in the other two cases were co-infection of V-leu and P-ala subtypes (Table 2). The sequence variations of the V-leu subtype in EBVaGCs in GRC were the same as those in EBVaGCs in CGC.

### V-val Subtype was Prevalent in TWs of Healthy EBV Carriers

**Figure 3:**
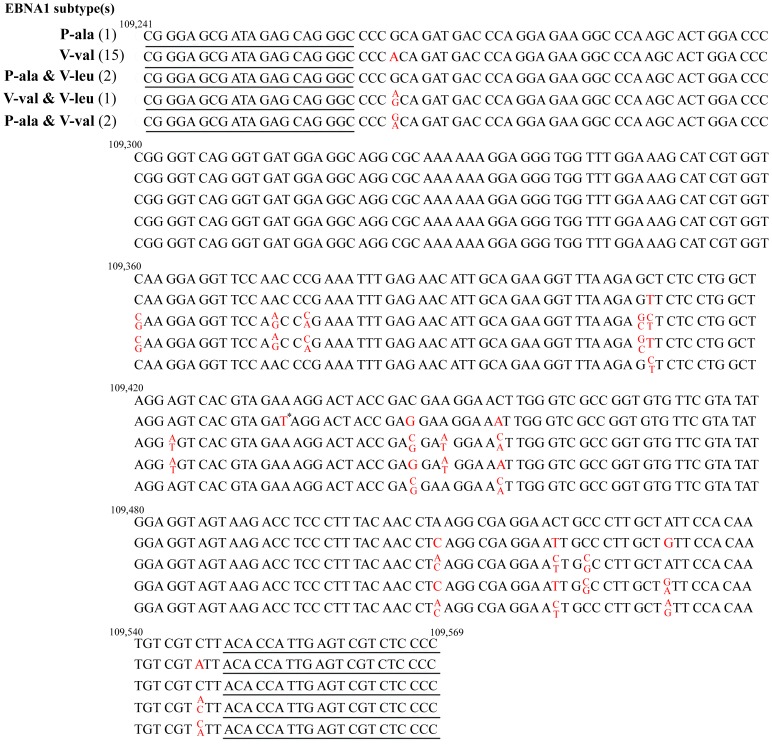
Nucleotide sequences of EBNA1 in TWs of healthy EBV carriers. The representative nucleotide sequences of EBNA1 (nucleotides 109,241∼109,569) in TWs of healthy EBV carriers are shown. Numbers across the top correspond to the nucleotide positions. The primer sequences (nucleotides 109,241∼109,260 and nucleotides 109,549∼109,569) are underlined. The numbers in the parentheses denote the amount of identical sequences. Mutated nucleotides with respect to P-ala subtype (the B95.8 prototype) are indicated in red. “*” indicated that an A→T mutation at nucleotide 109,434 was detected in only one case of the V-val subtype.

The EBNA1 fragment was successfully amplified in 21 (24.1%) of 87 TW samples from healthy donors. Sequencing results revealed that V-val subtype was the most predominant (85.7%, 18/21) in TWs of healthy EBV carriers. Among the 18 cases, 15 (83.3%) cases harbored a single V-val subtype sequence, while the other 3 (16.7%) cases occurred as mixed infection of V-val and P-ala or V-leu subtypes. The distribution of EBNA1 subtypes in TWs of healthy EBV carriers is showed in Table 2. All but one of the sequences were identical to those in EBVaGCs in CGC. However, one case of the V-val subtype exhibited an additional point mutation at position 109,434 (A→T), which resulted in an amino acid change at codon 495 (E→D). The nucleotide sequences of EBNA1 in TWs of healthy EBV carriers are shown in Figure 3.

### Distribution of EBNA1 Subtypes in EBVaGC in CGC and TWs of Healthy EBV Carriers

The V-val subtype was the most predominant in both EBVaGC in CGC and TWs of healthy EBV carriers, accounting for 53.6% (15/28) and 85.7% (18/21), respectively. The difference of V-val subtype in EBVaGC in CGC and TWs of healthy EBV carriers was not statistically significant (*p* = 0.102; OR: 0.252, 95%CI: 0.048–1.315). The P-ala subtype was found in 12 of 28 (42.9%) EBVaGCs in CGC, and in 5 of 21 (23.8%) TWs of healthy EBV carriers. The distribution of P-ala subtype in EBVaGC in CGC and TWs of healthy EBV carriers was not statistically significantly different, either (*p* = 0.487; OR: 1.654, 95%CI: 0.400–6.838). Similarly, the distribution of V-leu subtype in EBVaGC in CGC and TWs of healthy EBV carriers was not statistically significantly different (32.1%, 9/28 vs. 14.3%, 3/21; *p* = 0.512; OR: 1.727, 95%CI: 0.337–8.855).

## Discussion

In the present study, EBNA1 was expressed in 93.3% (42/45) of the EBVaGC cases in CGC and 87.5% (7/8) of the EBVaGC cases in GRC by immunohistochemistry (IHC). This is consistent with the previous studies, in which EBNA1 was almost expressed in all EBVaGC cases, irrespective of the detection methods used, either by IHC/immunofluorescence to detect the protein or by reverse transcription-PCR (RT-PCR) to detect the mRNA [39], [40], [41], [42]. There was no correlation between EBNA1 expression and the clinicopathologic parameters of EBVaGC, which include the age and sex of the patient, the location, histology and stage of the tumor.

The present study not only detected the expression of EBNA1 in EBVaGC but also for the first time investigated the variations of EBNA1 in EBVaGC in Guangzhou, southern China, which is an endemic area of NPC. Carboxyl-terminal fragment (codons 431∼540) of EBNA1 was successfully amplified and sequenced in 28 EBVaGCs in CGC, 4 EBVaGCs in GRC and 21 TWs of healthy EBV carriers. The sequencing results showed that V-val subtype was the most predominant (53.6%, 15/28) in EBVaGCs in CGC, followed by P-ala (42.9%, 12/28) and V-leu (32.1%, 9/28) subtypes. In EBVaGCs in GRC, V-leu subtype was detected in all 4 (100%) cases. In TWs of healthy EBV carriers, V-val subtype was also predominant (85.7%, 18/21). The distribution of EBNA1 subtypes in EBVaGC in CGC and TWs of healthy EBV carriers was not statistically significantly different.

In the present study, V-val subtype was predominant in EBVaGC in CGC in Guangzhou, southern China, which is similar to that in TWs of healthy EBV carriers in the same area. Wang et al. [27] also found that V-val subtype was prevalent in EBVaGC and TWs of healthy donors in Shangdong Province, northern China, accounting for 78.1% (32/41) and 61.8% (34/55), respectively. In Japan, all 25 (100%) cases of EBVaGC harbored the V-val subtype, while in America, however, P-thr subtype was predominant, accounting for 52.9% (9/17) of the EBVaGC cases [26]. It is known that in healthy donors, V-val subtype is predominant in Asia, while P-thr subtype is common in North America [43]. Thus, the EBNA1 subtypes in EBVaGC were similar to those in the background population from the same areas, suggesting that the EBV strains in EBVaGC might originate from the viral strains prevalent within the background population, and the variations of EBNA1 in EBVaGC were due to geographic-associated polymorphisms rather than EBVaGC-associated mutations.

Other than EBVaGC, in other EBV-associated neoplasms, V-val subtype was predominant in individuals from Asian regions, while P-thr and P-ala subtypes were common in Europe and North America, and P-thr and V-leu subtypes were predominant in Africa and South America [43]. It is known that NPC, EBVaGC and nasal NK/T-cell lymphoma are more prevalent in Asia than in other part of the world [44], thus, it is likely that V-val subtype might be more aggressive than other subtypes. Recently, Mai et al. [45] and Do et al. [46] demonstated that V-val subtype had functional advantage and higher transcriptional activity than P-ala subtype (the B95.8 prototype). As compared with the prototype P-ala, V-val subtype showed 7 amino acid substitutions (codons 439, 487, 499, 502, 524, 528 and 533) with respect to the carboxyl-terminus of EBNA1. Most of these amino acid alterations locate in the function domains of EBNA1, including dimerization domain (aa 501∼532), DNA binding domain (aa 459∼487), and transactivation domain (aa 450∼641) [20], [21], [22], [23]. Therefore, these sequence variations may influence the DNA binding, thus involving in DNA replication and transcription, leading to changes of EBNA1’s function. Besides EBV infection, both geography and genetics may also influence disease risk. A study of Americans with Japanese ancestry, mostly born in Hawaii, reported 19 of 187 (10.2%) gastric cancer cases to be EBV-associated [47]. The observed percentage of EBVaGC was intermediate between Japanese (6.9%) [48] and Caucasians in Los Angeles (16%) [49], suggesting that the frequency of EBVaGC may be affected by environmental factors. By an entire genomic scan linkage analysis, Feng et al. localized an NPC susceptibility locus to chromosome 4p12∼4p15 in a subset of families at high risk of NPC from Guangdong Province, southern China [50]. Therefore, the interplay of EBV infection, genetic susceptibility and environmental factors would together contribute to a higher risk of EBV-associated neoplasms development.

In the present study, the EBNA1 variations were for the first time determined in 4 (50%) of the 8 EBVaGCs in GRC. V-leu subtype was predominant, since it was detected in all 4 (100%) EBVaGCs in GRC, although 2 cases occurred as mixed infection with P-ala subtype. This is different from the predominant V-val subtype in EBVaGC in CGC. As compared with V-val subtype, V-leu subtype showed 10 amino acid substitutions (codons 439, 471, 475, 476, 487, 492, 500, 525, 528 and 533) with respect to the carboxyl-terminus of EBNA1. Whether these variations could affect the function of the EBNA1 protein and result in differences between V-leu and V-val subtypes is uncertain and needs to be further investigated.

The principal difference between the remnant stomach and the non-operative stomach is the mechanical and chemical injuries of the remnant gastric mucosa due to the operation and the duodenogastric reflux. Besides, the duodenogastric reflux causes an increasing pH-value, resulting in growth of bacteria in the gastric stump, which thus leads to changes of the microenvironment within the gastric stump [31]. The injuries of remnant gastric mucosa and changes of the microenvironment within the gastric stump will lead to inflammation in the remnant stomach, which may facilitate EBV entering the remnant gastric mucosa epithelia and eventually promote the development of EBVaGC [35]. Given that V-leu subtype was predominant in EBVaGC in GRC, it is suggested that V-leu might be a subtype that adapts particularly well to the microenvironment within the gastric stump and enters the remnant gastric mucosa epithelia easily. However, this hypothesis needs to be further investigated.

In conclusion, the EBNA1 polymorphisms in EBVaGC in Guangzhou were similar to those in EBVaGC in northern China and Japan, but were different from those in EBVaGC in America, which suggests that the variations of EBNA1 in EBVaGC represent geographic-associated polymorphisms rather than tumor-specific mutations. In addition, the sequence variations of EBNA1 in EBVaGC were similar to those in TW of healthy EBV carriers in the same area, which seems to suggest that the EBV strains in EBVaGC originate from the viral strains prevalent within the background population. Moreover, the EBNA1 variations in EBVaGC in GRC were different from those in EBVaGC in CGC. Further studies are required to investigate the functional and immunological impact of EBNA1 sequence variations and to evaluate their possible significance, which could also be helpful to clarify the association of EBNA1 subtypes and EBV-associated malignancies, and provide important insights to the roles of EBV in the pathogenesis of EBV-associated malignancies.

## Methods

### Ethics Statement

This study was approved by the Clinical Research Ethics Committee of the Third Affiliated Hospital, Sun Yat-sen University. Written informed consents were taken from all the patients and healthy donors and ethical guidelines under Declaration of Helsinki were followed.

### Subjects

Fifty-three cases of EBVaGC, including forty-five cases of CGC and eight cases of GRC, which were determined by EBV-encoded small RNA-1 (EBER1) *in situ* hybridization [7], [35], were included in the present study. All the GRC cases had received partial gastrectomy for reasons of benign diseases, including gastric ulcer (5 cases) and duodenal ulcer (3 cases). All cases were collected in the Second and Third Affiliated Hospitals of Sun Yat-sen University and the Guangzhou First Municipal People’s Hospital, Guangzhou, southern China, during the period from January 1, 2000 to December 31, 2006. The patients were all Guangzhou natives. All the tumor specimens were obtained from surgical resection cases. Paraffin-embedded tissues and clinicopathologic data including age and sex of the patient as well as the location, macroscopic type, invasion depth and lymphatic and hematogenous metastases of the tumor, were retrieved from the three Departments of Pathology.

TW samples of 87 healthy donors were collected in the Third Affiliated Hospital of Sun Yat-sen University. The donors were all Guangzhou native. Among them, 51 (58.6%) donors were male, while the remaining 36 (41.4%) donors were female. The mean age was 46.6±17.1 years (range: 22–73 years, median: 52 years). TW samples were collected by gargling with 15 ml of phosphate buffered saline (PBS).

### Histologic Examination

Histology of the gastric carcinomas was classified as intestinal- and diffuse-type according to the Lauren classification [51]. Subsequently, the intestinal-type gastric carcinomas were subclassified as papillary adenocarcinoma (pap), well-differentiated tubular adenocarcinoma (tub1), or moderately differentiated tubular adenocarcinoma (tub2); the diffuse-type gastric carcinomas were subclassified as solid poorly differentiated adenocarcinoma (por1), non-solid poorly differentiated adenocarcinoma (por2), signet ring cell carcinoma (sig), or mucinous carcinoma (muc) according to the Japanese classification [52]. The macroscopic type, location, invasion depth and staging of the tumors were also classified according to the Japanese classification [52].

### Immunostaining for EBNA1

The immunostaining for EBNA1 was performed using the two-step EnVision immunohistochemical procedure (Dako, Denmark) as previously described [7]. The monoclonal antibody against EBNA1 (Millipore, Billerica, MA) was applied. The signals were visualized with DAB, and the slides were counterstained with Mayer’s hematoxylin. The known EBNA1-positive NPC tissues were used as positive controls. PBS other than the primary antibodies was used as the negative control. Tumors were considered positive if 10% or more of the neoplastic cells were stained.

### DNA Extraction

DNA was extracted from formalin-fixed, paraffin-embedded tumor tissues and TWs of healthy donors using the NucleoSpin® Tissue Kit (MACHEREY-NAGEL GmbH & Co. KG, Germany) according to the manufacturer’s instructions. The extracted DNA sample was dissolved in 100 μl of TE buffer. Paraffin blocks without any samples were used as negative controls. DNA extracted from the formalin-fixed, paraffin-embedded EBV-positive cell line B95.8 [17] was used as positive control.

### Polymerase Chain Reaction

Polymerase chain reaction (PCR) was performed with 2 μl of DNA in a 50 μl total reaction mixture containing 10 mM Tris-HCl (pH 8.0), 50 mM KCl, 1.5 mM MgCl_2_, 200 μM dNTP, 0.5 μM of each primer and 1.25 U Taq Polymerase (TaKaRa, Dalian, China). The primer sets are as follows: EBNA1-F: 5′-CGGGAGCGATAGAGCAGGGC-3′ (B95.8 coordinate 109,241–109,260); EBNA1-R: 5′-GGGGAGACGACTCAATGGTGT-3′ (B95.8 coordinate 109,549–109,569). The PCR product was 329 bp. The amplification protocol was one cycle at 94°C for 5 min, followed by 40 cycles of denaturation at 94°C for 1 min, annealing at 57°C for 1 min and elongation at 72°C for 90 sec, and a final extension at 72°C for 10 min. The amplified mixtures were visualized by electrophoresis in a 1.5% agarose gel stained with 0.5 μg/ml of ethidium bromide and photographed under UV light.

### DNA Sequencing

The products of the PCR reaction were extracted and purified using QIAquick PCR Purification Kit (QIAGEN, Hilden, Germany). The purified PCR products were then subjected to DNA sequencing. Cycle sequencing was carried out using the ABI PRISM BigDye Terminator Cycle Sequencing Ready Reaction Kit (PE Applied Biosystems, Foster City, CA, USA) according to the manufacturer’s manual. Sequence analysis was performed on the 3730×l DNA Analyzers, and the data were analyzed with Seq Ed. software (PE Applied Biosystems). All sequences were performed bi-directionally. PCR product of B95.8 cells was simultaneously sequenced to ensure the integrity of the run. Mixed infection was defined if multiple signals occurred at the same nucleotide positions. The sequencing results were then compared with the B95.8 prototype strain (GenBank Accession No.: V01555) as well as other published EBV strains using the BLAST software from the NCBI website (http://blast.ncbi.nlm.nih.gov/Blast.cgi) to determine the difference in the nucleotide sequences.

### Determination of EBNA1 Subtypes

The EBNA1 subtypes were determined based on the signature amino acid (AA) residue 487 (coded by nucleotides 109,408∼109,410) as well as particular amino acid alterations in other sites, proposed by Bhatia *et al*. [24] and Gutiérrez *et al*. [25]. P-ala was determined when the sequence was identical to B95.8 prototype. V-val was confirmed when C signal changed to T at nucleotide 109,409 as well as particular nucleotides alterations in other sites. V-leu was confirmed when GCT signals changed to CTT at nucleotides 109,408∼109,410 with particular variations at other sites. Co-infection of V-val and V-leu was defined when G and C signals occurred simultaneously at nucleotide 109,408, and TT signals at nucleotides 109,409∼109,410. If C and T signals occurred simultaneously at nucleotide 109,409, and G and T signals at nucleotides 109,408 and 109,410, respectively, it was determined as co-infection of P-ala and V-val. If A and C signals occurred simultaneously at nucleotide 109,408, and C and T signals at nucleotide 109,409, it was determined as co-infection of V-leu and P-thr. If G and C occurred at nucleotide 109,408, and C and T at nucleotide 109,409, it was determined as co-infection of P-ala and V-leu, or mixed-infection of P-ala, V-leu and V-val, which was distinguished by alterations in other sites.

### Statistical Analysis

Fisher’s exact tests or Student’s *t* tests were used to compare the clinicopathologic characteristics of EBVaGC in CGC and GRC. Binary logistic analyses were conducted to compare the distributions of EBNA1 subtypes in EBVaGC in CGC and TWs of healthy EBV carriers. Sex and age were included in the logistic model as covariates. Sex was analyzed as a categorical variable, while age was analyzed as a continuous variable. Maximum likelihood estimates of odds ratios (ORs) and their corresponding 95% confidence intervals (95% CIs) were calculated from the multivariable logistic regression model. The results were considered to be statistically significant at a *p*-value of less than 0.05. All the *p*-values presented in the present study are two-sided.

## Supporting Information

Table S1 Clinicopathologic data and EBNA1 subtype(s) of the 28 EBVaGC cases.
(DOC)
