# Supplementary material for: Variations of Epstein-Barr Virus Nuclear Antigen 1 in Epstein-Barr Virus-Associated Gastric Carcinomas from Guangzhou, Southern China
Source: PLoS One. 2012 Nov 26;7(11):e50084. doi: 10.1371/journal.pone.0050084 (PMC3506544; doi:10.1371/journal.pone.0050084)
Supplement: Table S1 — Clinicopathologic data and EBNA1 subtype(s) of the 28 EBVaGC cases. (DOC) [file pone.0050084.s001.doc]

**Table S1. Clinicopathologic data and EBNA1 subtype(s) of the 28 EBVaGC cases.**

| **Gender** | **Age (years)** | **Macroscopic type^*^** | **Location^*^** | **Histology (Lauren classification)** | **Histology (Japanese classification)**^†^ | **Staging (TNM)****^*^** | **EBNA1 subtype(s)** |
| --- | --- | --- | --- | --- | --- | --- | --- |
| Male | 40 | 2 | Cardia | Diffuse | por2 | 3a (T3N1M0) | P-ala/V-leu |
| Male | 59 | 2 | Antrum | Diffuse | por1 | 3a (T3N1M0) | V-leu |
| Male | 52 | 3 | Antrum | Diffuse | por2 | 4 (T3N3M0) | P-ala/V-leu |
| Male | 45 | 4 | Whole**^‡^** | Diffuse | por1 | 4 (T4N1M0) | V-val/V-leu |
| Male | 37 | 3 | Body | Diffuse | por1 | 2 (T2N1M0) | V-val |
| Male | 35 | 2 | Antrum | Intestinal | tub2 | 4 (T3N3M0) | P-ala |
| Female | 23 | 1 | Cardia | Diffuse | por2 | 3a (T3N1M0) | V-val |
| Female | 70 | 4 | Whole**^‡^** | Diffuse | por2 | 2 (T3N0M0) | V-val |
| Male | 53 | 2 | Body | Intestinal | tub2 | 3b (T3N2M0) | V-val |
| Male | 42 | 3 | Antrum | Diffuse | por2 | 1b (T2N0M0) | P-ala/V-val |
| Male | 40 | 1 | Cardia | Diffuse | por1 | 3b (T3N2M0) | V-val |
| Female | 52 | 1 | Cardia | Diffuse | por1 | 3a (T3N1M0) | P-ala |
| Male | 75 | 3 | Cardia | Diffuse | por2 | 3a (T4N0M0) | P-ala |
| Male | 59 | 2 | Antrum | Diffuse | por1 | 3a (T3N1M0) | V-leu |
| Male | 58 | 3 | Body | Diffuse | por2 | 4 (T3N3M0) | P-ala |
| Female | 73 | 3 | Body | Intestinal | tub2 | 3a (T3N1M0) | P-ala |
| Male | 49 | 3 | Antrum | Diffuse | por2 | 3a (T3N1M0) | P-ala/V-val |
| Male | 58 | 1 | Cardia | Intestinal | tub2 | 4 (T3N3M0) | P-ala |
| Male | 71 | 3 | Antrum | Diffuse | por2 | 3a (T3N1M0) | P-thr/V-leu |
| Male | 46 | 1 | Cardia | Diffuse | por2 | 2 (T3N0M0) | V-val |
| Male | 38 | 3 | Cardia | Diffuse | por2 | 3a (T3N1M0) | P-ala |
| Female | 53 | 1 | Body | Diffuse | por2 | 3b (T3N2M0) | V-val/V-leu |
| Male | 55 | 2 | Body | Diffuse | por2 | 3b (T3N2M0) | V-val/V-leu |
| Male | 39 | 2 | Cardia | Intestinal | tub2 | 4 (T4N2M0) | V-val |
| Male | 44 | 3 | Body | Diffuse | por2 | 4 (T3N3M0) | V-val |
| Male | 34 | 4 | Antrum | Diffuse | por2 | 3b (T3N2M0) | V-val |
| Male | 59 | 3 | Cardia | Diffuse | por2 | 4 (T3N3M0) | P-ala/V-leu |
| Male | 48 | 3 | Body | Diffuse | por2 | 2 (T2N1M0) | V-val |

^*^Japanese classification. ^†^tub2: moderately differentiated tubular adenocarcinoma; por1: solid poorly differentiated adenocarcinoma; por2: non-solid poorly differentiated adenocarcinoma. ^‡^Cases involved the whole stomach.
